# Changes in structure and assembly of a species-rich soil natural community with contrasting nutrient availability upon establishment of a plant-beneficial *Pseudomonas* in the wheat rhizosphere

**DOI:** 10.1186/s40168-023-01660-5

**Published:** 2023-09-29

**Authors:** Daniel Garrido-Sanz, Senka Čaušević, Jordan Vacheron, Clara M. Heiman, Vladimir Sentchilo, Jan Roelof van der Meer, Christoph Keel

**Affiliations:** Department of Fundamental Microbiology, University of Lausanne, CH-1015 Lausanne, Switzerland

**Keywords:** Microbiome, Nutrient availability, Plant-beneficial inoculant, Inoculant proliferation, Rhizosphere, Wheat, Bulk soil, Microbial communities, *Pseudomonas*, Bacterial competition

## Abstract

**Background:**

Plant-beneficial bacterial inoculants are of great interest in agriculture as they have the potential to promote plant growth and health. However, the inoculation of the rhizosphere microbiome often results in a suboptimal or transient colonization, which is due to a variety of factors that influence the fate of the inoculant. To better understand the fate of plant-beneficial inoculants in complex rhizosphere microbiomes, composed by hundreds of genotypes and multifactorial selection mechanisms, controlled studies with high-complexity soil microbiomes are needed.

**Results:**

We analysed early compositional changes in a taxa-rich natural soil bacterial community under both exponential nutrient-rich and stationary nutrient-limited growth conditions (i.e. growing and stable communities, respectively) following inoculation with the plant-beneficial bacterium *Pseudomonas protegens* in a bulk soil or a wheat rhizosphere environment. *P. protegens* successfully established under all conditions tested and was more abundant in the rhizosphere of the stable community. Nutrient availability was a major factor driving microbiome composition and structure as well as the underlying assembly processes. While access to nutrients resulted in communities assembled mainly by homogeneous selection, stochastic processes dominated under the nutrient-deprived conditions. We also observed an increased rhizosphere selection effect under nutrient-limited conditions, resulting in a higher number of amplicon sequence variants (ASVs) whose relative abundance was enriched. The inoculation with *P. protegens* produced discrete changes, some of which involved other *Pseudomonas*. Direct competition between *Pseudomonas* strains partially failed to replicate the observed differences in the microbiome and pointed to a more complex interaction network.

**Conclusions:**

The results of this study show that nutrient availability is a major driving force of microbiome composition, structure and diversity in both the bulk soil and the wheat rhizosphere and determines the assembly processes that govern early microbiome development. The successful establishment of the inoculant was facilitated by the wheat rhizosphere and produced discrete changes among other members of the microbiome. Direct competition between *Pseudomonas* strains only partially explained the microbiome changes, indicating that indirect interactions or spatial distribution in the rhizosphere or soil interface may be crucial for the survival of certain bacteria.

Video Abstract

**Supplementary Information:**

The online version contains supplementary material available at 10.1186/s40168-023-01660-5.

## Background

The use of bacterial inoculants in agriculture has emerged as an ecological and sustainable alternative to chemical fertilizers and pesticides. These inoculants have the ability to promote plant growth and health through different mechanisms, including solubilization of nutrients [1], protection against abiotic stresses such as drought or salinity [2, 3], enhancing the plant development by production of phytohormones [4], antagonizing soilborne phytopathogens [5] or killing pest insects [6, 7]. Despite these properties, field application of bacterial inoculants faces many challenges, notably their inability to efficiently compete and establish within the resident root microbiota during the time needed to exert their beneficial effect [8], often requiring multiple inoculations to ensure their function [9].

The assembly of the rhizosphere microbiome starts early during plant development with the release of root exudates containing carbon-rich compounds that create a niche capable of supporting bacterial growth in high numbers [10, 11]. These root exudates produce a rapid change in the bulk soil microbiome (not influenced by roots) towards a more specialized and metabolically active community (i.e. the rhizosphere microbiome) whose members are able to exploit the resources of the new niche [12, 13]. The rhizosphere microbiome is in part determined by the composition of the plant root exudates, which vary across genotypes, plant-developmental stages or in response to environmental changes [14, 15]. In addition, root exudates also contain multiple plant secondary metabolites (e.g. flavones, benzoxazinoids and coumarins) involved in microbiome signalling processes by facilitating or inhibiting the growth of susceptible bacteria, thus impacting microbiome assembly and functioning [16–18]. The interactions between the microbiome members will also determine the rhizosphere microbiome structure and composition [19]. Bacteria can compete with other bacteria for resources or space using contact-dependent or diffusible weaponry [20–22] or display positive, mutualistic relationships, which are often found dominating the rhizosphere [23, 24].

The effective establishment of plant-beneficial bacterial inoculants in the rhizosphere microbiome is dependent on their ability to proliferate under the conditions and competing factors of the rhizosphere. Notably, a poor adaptability of the inoculant to varying environmental conditions, such as soil pH [25], soil humidity or regional climate, might result in a suboptimal and/or transient colonization [8, 26, 27]. In addition, the fate of the inoculant also depends on biotic interactions, which are often intricate, expanding to multiple trophic levels and higher orders of interactions [28–30]. These can include complex networks involving competition, cooperation [23] or interaction with other microbes. Also, grazing by phagotrophic protists [31] and infection by bacteriophages [32, 33] can prevent an effective colonization of the rhizosphere microbiome by inoculants. An important factor governing inoculant proliferation is the availability of nutrients in the rhizosphere environment to support the community growth and development [34–36], which will be different from the bulk soil nutrients, often scarce, limiting bacterial growth and soil microbial processes [37]. Compositional changes of such microbiomes in response to the proliferation of an inoculant could provide important information that will improve our understanding of how plant-beneficial bacterial inoculants interact with the rhizosphere microbiome.

Soil and rhizosphere microbiomes are considered the most diverse ecosystems on earth [38], harbouring hundreds of genotypes. This challenges the use of natural microbiomes for elucidating invasion strategies of plant-beneficial inoculants [26, 27, 39–42]. Replicable and simplified communities are often obtained in standardized systems through top-down approaches to further study how they react to stressors, including the introduction of new community members [43–45]. The extreme of such reductionistic approaches is the study of individual bacterial isolates or of synthetic communities. They can be of great importance for investigating mechanistic effects that however might not always be observed under natural conditions. This challenge might be eased by using species-rich natural communities directly derived from the environment [46, 47].

In the present study, we analysed the potential of the model plant-beneficial bacterium *Pseudomonas protegens* CHA0^T^ [5, 22, 48] to establish in the rhizosphere of wheat (*Triticum aestivum*) as a function of the physiological state of a reproducible, species-rich bulk soil natural bacterial community (NatCom) [46]. Two different community-growing states were evaluated based on nutrient availability to study differences of an actively growing community (i.e. by dilution of the NatCom and addition of nutrients) versus a stationary, nutrient-limited stable NatCom, with the aim to simulate two nutritional scenarios that occur in natural soils.

## Materials and methods

### Experimental design

A previously isolated and characterized soil natural bacterial community (NatCom) was grown and prepared in microcosms as detailed in [46]. Briefly, microcosms consisting of soil matrix (0.5–3 mm washed and sterile riverbank silt) and 10% (v/w) of soil extract (SE, autoclaved and filtered aqueous top forest soil solution, see [46] for details) as source of nutrients were inoculated with 10% (w/w) of the soil NatCom [46]. Community growth was tracked weekly by counting of colony-forming units (CFUs) on R2A medium (Sigma-Aldrich) for about 1 month until an apparent community stationary phase was achieved, reaching ~10^8^ CFUs g^−1^ of soil matrix. Two community states were chosen: (1) the growing NatCom and (2) the stable NatCom. For the growing NatCom, the community was diluted (10% (w/w)) into new soil matrix and soil extract as described above. This was considered the growing NatCom, as the community had access to new nutrients (i.e. soil extract) to grow. The stable NatCom corresponded to the above-mentioned NatCom in an overall community stationary growth phase, which had not been provided with new nutrients for a month. For each community, growing or stable, part was inoculated with *Pseudomonas protegens* CHA0^T^ tagged with mCherry (CHA0::attTn7-*mcherry*; gentamycin resistant (Gm^R^) [49]) to obtain 10^5^ CFUs g^−1^ of soil matrix, while the other part remained uninoculated. Cells of *P. protegens* CHA0^T^-*mcherry* (*P. protegens* CHA0 hereafter) were prepared from an overnight culture in nutrient yeast broth (NYB, per 1 L: 25 g of nutrient broth no. 2, Oxoid, and 5 g of yeast extract, Oxoid), supplemented with gentamycin sulphate (Gm; 10 µg mL^−1^). Cells were harvested by centrifugation at 5500 × *g* for 5 min and washed with mineral medium (MM: for 1 L; 1 g of NH_4_Cl; 3.49 g of Na_2_HPO_4_·2H_2_O; 2.77 g of KH_2_PO_4_, pH adjusted with NaOH 10 M to 6.8, autoclaved at 121 °C for 20 min) supplemented with 0.1 mM succinate. Cells were diluted to a final optical density at 600 nm (*OD*_600_) of 0.1 (~10^7^ CFUs mL^−1^). One millilitre of this cell suspension was thoroughly mixed into 100 g of soil matrix.

Twenty grams of soil matrix containing the growing NatCom or the stable NatCom, inoculated or not with *P. protegens* CHA0, was transferred into sterile 3.3 borosilicate glass tubes (diameter Ø25 mm × 200 mm, Fisher Scientific), and, for the rhizosphere samples, a surface-disinfected and pregerminated seed of wheat (*Triticum aestivum*, cv. Arina) was transferred into the system in sterile conditions. Tubes without wheat were considered as bulk soil. All tubes were closed with sterile Magenta^™^ 2-way polypropylene caps (Sigma-Aldrich) to allow gas exchange. The tubes were kept in a Percival PGC-7L2 plant growth chamber at 22/18 °C, 16/8-h light/dark photoperiod (light intensity, 160 μE m^−2^ s^−1^), with a relative chamber humidity of 70% until tubes were sampled. Wheat seeds were surface disinfected in 4% NaClO for 15 min and then washed ten times with 40 mL of sterile distilled water. Disinfected seeds were placed on 1.5% (w/v) agar (agar bacteriological no. 1, Oxoid) plates and germinated for 2 days in darkness in the growth chamber before transferring them into the tubes. Sixteen tubes per condition and sampling time (see below) were prepared.

Microcosms were sampled 1, 5, 7 and 9 days post inoculation (dpi). Per sampling time, plants were carefully removed from the tubes, and roots were gently shaken to remove loosely adhering soil matrix particles. Roots with the adhering soil matrix were then cut off at their point of emergence from the seed. This was considered to be the rhizosphere, which also encompasses the rhizoplane. The rhizospheres of four plants were pooled and weighed. Five grams of four individual bulk soil samples was also combined into one and weighed. Four replicates per condition and timepoint were carried out (i.e. a total of 16 individual microcosms per condition and sampling time, pooled by four). Twenty millilitres of MM without carbon source was added to each pool of samples and vortexed for 15 min. Samples were then centrifuged at 300 × *g* for 1 min to pellet soil matrix debris. Then, 10 mL of the supernatants was collected and centrifuged at 7000 × *g* for 15 min to pellet the bacterial cells, which were frozen at −20 °C until DNA extraction.

Fresh weight was assessed for each pool of four shoots or roots, which were at first washed to remove adhering soil matrix particles. The individual length of shoots was measured, and average values calculated per pool of four. To assess the dry weight, roots and shoots were then fully dried for 2 weeks at 37 °C.

### DNA extraction, library preparation, sequencing and ASVs identification

Total DNA extractions were carried out using the DNeasy PowerSoil Pro kit (Qiagen) following the manufacturer’s instructions, and DNA concentrations were measured using the Qubit^™^ dsDNA BR assay kit (Invitrogen). DNA samples were stored at −20 °C. To prepare the small ribosomal subunit (16S rRNA) gene amplicon libraries, 10 ng of extracted DNA per sample was used to amplify the V3–V4 region of the 16S rRNA gene following the Illumina 16S Metagenomic Sequencing Library protocol [50] using the A Nextera XT index kit (v2, Illumina) for indexing. Samples were then quantified and pooled in equal amounts for sequencing. Pooled samples were spiked with 25% PhiX control DNA and paired-end sequenced at the Lausanne Genomic Technologies Facility (Lausanne, Switzerland), using an Illumina MiSeq v3 instrument running for 300 cycles.

The obtained raw sequence reads were quality filtered and trimmed using fastp v0.23.2 [51] with adaptor autodetection and default parameters. Quality-filtered reads were processed following the DADA2 v1.16.0 [52] pipeline [53]. Briefly, after plotting read-quality scores, sequences were truncated when the average mean quality score dropped below 28 and using a maximum number of expected errors of 2 and 3 for forward and reverse reads, respectively. After merging error-corrected reads into sequences, the distribution of lengths was inspected, and those with a length between 401 and 428 nucleotides were kept. The rest of the steps and parameters were kept as in the pipeline specified above. Sequences obtained after chimera removal were considered as amplicon sequence variants (ASVs). The number of surviving sequences per step throughout the pipeline are detailed in Supplementary Table S1. Taxonomy was assigned using the SILVA nonredundant (nr) database, v138 [54] at 99% sequence identity. ASVs were aligned with MAFFT v7.508 [55] to then construct a maximum-likelihood (ML) phylogeny using IQ-TREE v2.2.0.3 [56] with best-fitting nucleotide substitution automatic model detection and 1000 ultrafast bootstrap replicates.

### Diversity and composition analyses

ASV sequences, taxonomy, metadata and phylogeny were imported into phyloseq v1.42.0 R package [57]. ASVs classified as mitochondria or chloroplasts were removed, and a prevalence filtering threshold of 0.5% across samples was applied, resulting in a total of 1509 ASVs. Relative abundances at the bacterial class taxonomic rank were analysed and represented within the 100 most abundant ASVs, using the *plot_bar* function within phyloseq R package. Observed ASVs and the Shannon diversity index were calculated and plotted with the *plot_richness* phyloseq R function. Phylogenetic diversity (Faith’s PD) was calculated using the *pd* function within the picante v1.8.2 R package [58]. Nonmetric multidimensional scaling (NMDS) analyses were performed with the *ordinate* phyloseq R function by first transforming relative abundances into a Bray-Curtis dissimilarity matrix using the *vegdist* function within the vegan v 2.6–4 R package [59] and a *k* = 2. Bray-Curtis dissimilarities were also used for a hierarchical clustering by complete-linkage and heatmap representation using the heatmap function within the ComplexHeatmap v2.9.2 R package [60].

### Null community model for assembly processes

The β-nearest taxon index (βNTI) and Raup-Crick-based Bray-Curtis (RC_bray_) were used to determine the contribution of deterministic and stochastic assembly processes as previously described [61, 62]. Briefly, the βNTI measures the degree of deviation of the β-mean-nearest taxon distance (βMNTD) from the null expectations based on 1000 random shuffles of ASVs across the phylogenetic tree. Values of |βNTI| > 2 indicate deterministic selection, which can be further partitioned into heterogeneous (βNTI > 2) or homogenous (βNTI < 2) selection. While heterogeneous selection implies that selective pressures drive communities to divergent configurations, in homogeneous selection, these selective pressures push communities towards a common composition [63]. The remaining community pairs with |βNTI| < 2 indicate that the community is mainly assembled by stochastic processes. RC_bray_ can be used to further classify this stochastic fraction. Values of RCbray < −0.95 indicate communities influenced by homogenising dispersal (taxonomically more similar than expected; populations are capable of interactions, allowing members to freely exchange), while RCbray > 0.95 suggest dispersal limitation; populations are unable to mix leading to development via ecological drift. Values of |RCbray| < 0.95 indicate an undominated processes, where no single assembly process is capable of explaining variation [63]. The βNTI, RC_bray_ and assembly processes based on entire-community null models [61] were calculated using the *qpen.cm* function from the iCAMP v1.5.12 R package [64].

### Ecological association inference and differential abundance

Association inference between ASVs was evaluated using sparse inverse covariance estimation for ecological association inference (SPIEC-EASI) with the SpiecEasi v1.1.2 R package [65], using the Meinshausen and Bühlmann’s neighbourhood selection method [66]. ASVs count table was split by tested condition and grouping timepoints 5, 7 and 9. The top 150 most abundant ASVs were retrieved. A lambda minimum ratio of 1e^−1^, *nlambda* = 20, and 100 replicates were chosen. Networks were visualized using the ggnetwork v0.5.10 R package. Modularity was calculated via greedy optimization of modularity using the igraph v1.3.0 R package. Keystone taxa were identified using the scaled Kleinberg’s hub centrality score [67] and a threshold of 0.7.

Differential abundance of ASVs across samples was determined using DESeq2 v1.38.0 R package [68], which normalizes ASVs by the median ratio of ASV counts relative to geometric mean per ASV [69]. Significance was assessed using the Wald test and a local estimation of dispersion. ASVs with a |log_10_ fold change (FC)| ≥ 2.5 and an adjusted *p*-value (adj. *p*) < 0.01 were considered as significant.

### *Pseudomonas* sp. ASV1168 and ASV1173 isolation and constitutive tagging

Axenic cultures of different bacterial strains from the NatCom were previously obtained and characterized [46]. We screened these isolates to search for the closest relatives of ASV1169 and ASV1173 using blastn [70]. Two *Pseudomonas* isolates were identified, one isolate with a 16S rRNA gene sequence identity of 98.83 with both ASV1168 and ASV1169 (hereafter *Pseudomonas* sp. ASV1168) and another isolate with 99.06% sequence identity with ASV1173 (hereafter *Pseudomonas* sp. ASV1173) over the full ASV length (427 nucleotides). Strain ASV1168 was constitutively tagged with *gfp2* (ASV1168::attTn7-*gfp2*; Gm^R^, as previously described [71]), while a spontaneous rifampicin resistant (Rif^R^) mutant of strain ASV1173 was obtained by successive plating on nutrient agar (NA; Oxoid, CM0003) supplemented with 200 µg · mL^−1^ of rifampicin.

### Competition assays

*Pseudomonas protegens* CHA0^T^-*mcherry* Gm^R^, *Pseudomonas* sp. ASV1168-*gfp2* Gm^R^ and ASV1173-Rif^R^ were grown overnight in NYB supplemented with Gm 10 ng mL^−1^ or Rif 200 ng mL^−1^, respectively. Cultures were restarted in fresh media (1:100 (v/v)) until an exponential growth was achieved (*OD*_600_ = 0.4–0.6). Cells were collected by centrifugation at 5500 × *g* for 5 min at 4 °C, washed twice with MM and finally resuspended in SE. The *OD*_600_ was adjusted to 0.1 in SE, corresponding to *ca*. 10^−7^ CFUs mL^−1^. Serial dilutions were plated on R2A medium to calculate the initial CFU load. Cultures were mixed in pairs (1:1) or the three strains together (1:1:1). Two millilitres of the resulting cell mixtures was inoculated into 20 g of soil matrix (*ca*. 10^−5^ CFUs g^−1^, which also accounts for 10% SE (v/w)) contained in glass tubes as the bulk soil condition, and a pregerminated surface-disinfected wheat seed was added for the rhizosphere condition, as described above. Sixteen replicates per condition were carried out. Microcosms were maintained in the plant growth chamber under the same conditions as described above. After 9 days, samples were pooled by four, and bacteria were extracted from the rhizosphere or the bulk soil as described above. CFUs were counted following plating serial dilutions in triplicate on R2A medium. The competition index (CI) was calculated using the formula: *CI* = (Sx at *t*9–Sx at *t*0)/(Sy *t*9–Sy *t*0), where Sx and Sy represent the CFUs g^−1^ of soil matrix of the two strains being compared, recovered at the final timepoint (*t*9 days) or inoculated (*t*0), as previously described [22]. Plant growth was also evaluated as described above.

### Statistical analyses

Multiple comparisons between the relative abundance of different taxonomic ranks across community states, environments and inoculation pattern were assessed with the Kruskal-Wallis rank-sum test within the agricolae v1.4–5 R package [72], using normalized ASV counts by cumulative sum scaling (CSS) as previously described [73]. Post hoc tests were performed with the Fisher’s least significant difference (LSD) criterium, and *p*-values were corrected using the false discovery rate (fdr). Differences between Shannon diversity, pairwise Bray-Curtis dissimilarities and βNTI across community states, environments and inoculation pattern were calculated using the Wilcoxon rank-sum test within the *stat_compare_means* function of ggpubr R package. In addition, the effect of different variables on Bray-Curtis dissimilarities was assessed using PERMANOVA (permutational multivariate analyses of variance), with the *adonis2* function within the vegan R package, using 9999 permutations. Spearman correlation between Shannon diversity or pairwise Bray-Curtis dissimilarities and sampling time were calculated using the *stat_cor* function within the ggpubr R package, and data was fitted to a general additive model (GAM), using a *k* = 3 with the *geom_smooth* function with the ggplot2 v3.3.6 R package [74] and a confidence interval of 0.95.

## Results and discussion

### *Pseudomonas protegens* proliferates best in stable resident communities under accessibility to the rhizosphere niche

**Fig. 1:**
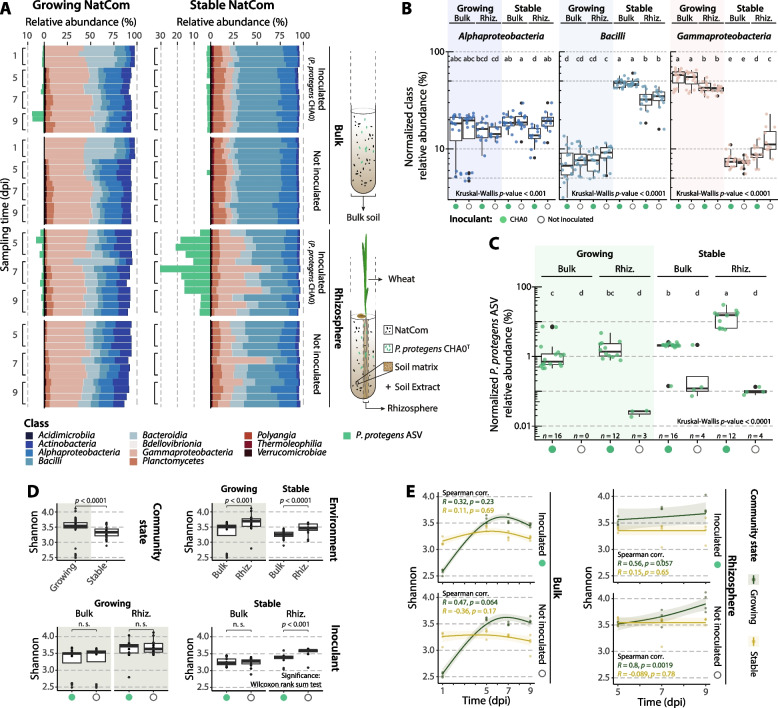
Composition and diversity of NatComs exposed to wheat roots and the inoculation of *Pseudomonas protegens*. **A** Relative abundance per replicate of the top 100 taxa at the bacterial class level in the growing or stable soil NatCom and of *P. protegens* CHA0 ASV (green bars, left) across sampling times (dpi, days post inoculation). **B** Differences in the CSS-normalized relative abundance of the three main classes Alphaproteobacteria, Bacilli and Gammaproteobacteria (see Supplementary Fig. 1 for all classes) or **C** *P. protegens* CHA0 ASV across samples. Samples taken at different sampling times are merged. For Gammaproteobacteria, *P. protegens* ASV was removed to avoid an artificial inflation of the class relative abundance. Significance based on Kruskal-Wallis rank-sum test with LSD post hoc analysis and *p*-value corrected by fdr. Different letters indicate significant differences between groups at *p*-value < 0.05. **D** Comparison of Shannon diversity in samples from different community states (growing or stable), environments (bulk soil or wheat rhizosphere (Riz.)) or inoculated with *P. protegens* CHA0 (represented by green dots) or not inoculated (empty dots). Significance based on Wilcoxon rank-sum test. Not significant (n.s.): *p*-value > 0.05. **E** Spearman correlation between the Shannon diversity index and sampling time in growing (green) or stable (yellow) community states. Correlation coefficient (*R*) and *p*-value (*p*) are indicated with the colour according to the growing state. Curves represent the general additive model (GAM) fit (average, line) and the 95% confidence interval (shadow)

We assessed the proliferation performance of the plant-beneficial inoculant *Pseudomonas protegens* CHA0 in response to plant roots (wheat) when exposed to a natural soil bacterial community (NatCom) in a growing versus a stable state. Simultaneously, we analysed the relative abundance of the different bacterial classes in the growing and stable NatComs. Overall, samples of the growing NatCom were dominated by Gammaproteobacteria (Fig. 1A; Supplementary Table S2), followed by Alphaproteobacteria, Bacteroidia, Actinobacteria and Bacilli. Conversely, the stable NatCom samples were dominated by Bacilli, followed by Alphaproteobacteria, Gammaproteobacteria, Bacteroidia and Planctomycetes. These bacterial classes are commonly found in soils worldwide [75, 76] and were also previously detected in the original soil NatCom used in this work [46]. However, the unusual higher abundance of Gammaproteobacteria in the growing community compared to other soils [75, 76] might indicate specific changes resulting from the initial high availability of nutrients (i.e. growing conditions), causing a dominance of fast-growing bacteria. These differences are better highlighted in a comparison of normalized relative class abundances between samples from the growing against the stable conditions (Fig. 1B, Supplementary Fig. S1). Notably, Acidimicrobiia, Bacilli, Planctomycetes, Polyangia and Thermoleophilia were significantly more abundant in the stable condition, while Actinobacteria and Gammaproteobacteria were more abundant in the growing community state. No differences in the relative abundance of Alphaproteobacteria and Bdellovibrionia were observed for most of the comparisons. Interestingly, bacterial classes whose relative abundances increased in stable, nutrient-limited conditions showed a decrease in the wheat rhizosphere (Fig. 1B, Supplementary Fig. S1). Although root exudates are rich in organic compounds [34], they also contain signalling molecules that could inhibit the growth of specific taxa, including Bacilli [16]. The opposite effect was observed for Gammaproteobacteria, whose abundances in the wheat rhizosphere increased under stable conditions compared to the corresponding bulk soil sample (Fig. 1B). In contrast, under growing conditions, most bacterial classes did not significantly differ in abundance in the wheat rhizosphere compared to the bulk soil. The exceptions were Acidimicrobiia and Actinobacteria, whose abundances increased (Fig. 1B). This might be due to a specific exploitation of root exudates. The differences observed between both community states (i.e. growing and stable) can likely be attributed to the initial access to nutrient niches under the growing condition, which allows the rapid growth of part of the population, resulting in the observed class differences.

Inoculation with *P. protegens* CHA0 did not significantly affect the relative abundances of the dominant bacterial classes. Notable exceptions were lower abundances of Actinobacteria, Alphaproteobacteria and Planctomycetes classes in the *P. protegens*-inoculated wheat rhizosphere under stable conditions (Fig. 1B, Supplementary Fig. S1). After removing *P. protegens* ASV counts from the Gammaproteobacteria calculations to avoid an artificial inflation, an increase in the abundance of this class was observed in the uninoculated wheat rhizosphere of stable conditions (Fig. 1B), suggesting a rhizosphere-specific effect. The fact that the largest changes in response to the plant-beneficial inoculant occurred within the wheat rhizosphere under stable, nutrient-limited conditions is probably the result of the selective effect of secreted root exudates. These contain nutrient-rich compounds that create a specific new niche within the otherwise niche-limited bulk soil, which is accessible to specific root-targeting microbiota [11, 48]. Indeed, the highest abundance of the ASV matching *P. protegens* CHA0 was detected in the rhizosphere of the stable NatCom (Fig. 1AC, average relative abundance across sampling times of 14.11%), which was significantly higher than in the bulk soil condition of the stable community (average relative abundance of 2.01%) and compared to the growing conditions (average relative abundance of 1.79% in the rhizosphere or 1.63% in bulk soil). The inoculant was therefore able to efficiently reach the rhizosphere microbiome and persisted for at least 9 dpi (Fig. 1C, Supplementary Table S2).

### *Pseudomonas protegens* proliferation alters the rhizosphere diversity under nutrient-limiting conditions

We next evaluated whether the diversity of the rhizosphere microbiome was influenced by the inoculant or by the nutrient availability. Growing NatComs exhibited significantly higher Shannon alpha diversity than stable ones (Fig. 1D), likely as a result of an initial higher availability in nutrients. Within the growing NatCom, the wheat rhizosphere significantly increased all diversity indexes (Shannon diversity, observed ASVs and Faith’s phylogenetic diversity), while the stable NatCom only showed increased Shannon diversity (Supplementary Fig. S2, Supplementary Table S3). This may point to a rhizosphere enrichment effect and can be attributed to the secretion of specific nutrients by the wheat roots [77]. Inoculation of *P. protegens* CHA0 did not result in significant differences in diversity, except in the rhizosphere of the stable condition, which showed a reduced Shannon diversity (Fig. 1D). However, the number of observed ASVs and the phylogenetic diversity remained constant here (Supplementary Fig. S2). This reduction in the Shannon diversity in the wheat rhizosphere of the stable NatCom is due to the population of *P. protegens* CHA0 (average relative abundance across timepoints of 14.11%, Fig. 1A, C), which proliferated in the niche that otherwise other NatCom bacteria would colonize. There was mostly no significant correlation of sampling time with Shannon diversity (Spearman correlation, *p*-value > 0.05; Fig. 1E), except for a positive correlation in the wheat rhizosphere under growing conditions (*R* = 0.8, *p*-value = 0.0019). This may be explained by the contribution of root exudates in addition to the nutrients contained in the soil extract.

Importantly, the inoculation with *P. protegens* CHA0 also produced a discrete increase in wheat growth compared to non-inoculated conditions (Supplementary Fig. S3). Mean weight values of fresh and dry shoots in the growing or stable conditions were higher in the inoculated samples, as were shoot lengths and root weights. The beneficial effect on plant growth of *P. protegens* CHA0 is in line with previous research reporting the strain’s growth-enhancing effects on different plant species [32, 78] and is related to its capacity to solubilize nutrients [79] and to synthesize phytohormones [80].

### Community succession is influenced by nutrient availability, wheat roots and *Pseudomonas protegens* proliferation

**Fig. 2:**
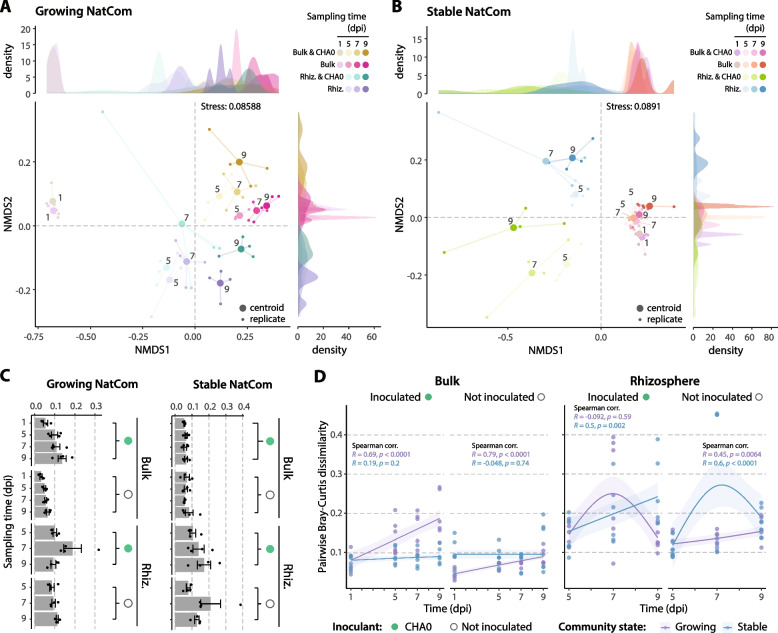
NatComs succession trajectories when exposed to wheat roots and the proliferation of *Pseudomonas protegens*. Nonmetric multidimensional scaling (NMDS) based on Bray-Curtis dissimilarities of the growing **A** or stable **B** NatCom samples, using a *k* = 2, and kernel density estimate of the replicate distribution per NMDS axis. Replicates (small dots) are connected to the centroid (big dot) by coloured lines according to samples and sampling times (dpi, days post inoculation). Numbers indicate sampling timepoint. **C** Inter-replicate consistency measured as the distance of each replicate to the centroid. Bars (±standard error) represent average values. Dots represent individual replicate values. **D** Spearman correlation between pairwise Bray-Curtis dissimilarities across samples and sampling time in growing (violet) or stable (blue) community states. Correlation coefficient (*R*) and *p*-value (*p*) are indicated. Curves represent the general additive model (GAM) fit (average, line) and the 95% confidence interval (shadow)

Community succession was evaluated based on Bray-Curtis dissimilarities. Samples from both community states showed different compositional succession over a period of 9 days (Fig. 2AB) and were clearly different based on hierarchical clustering (Supplementary Fig. S4). In the case of the growing NatCom, the inoculated *Pseudomonas* had an impact on both the bulk soil and wheat rhizosphere communities, leading to measurable diverging trajectories (PERMANOVA *p*-value = 0.0369). For the stable NatCom, all bulk soil samples clustered together, regardless of the sampling time or the inoculation pattern (Fig. 2B, Supplementary Fig. S4), whereas the corresponding wheat rhizosphere communities diverged, depending on inoculation with *P. protegens* CHA0. This shows that they are undergoing successional changes, coherent with the assembly of the root microbiome [81], the proliferation of *P. protegens* CHA0, or a combination of both. The finding that no differences in the trajectories of the bulk soil communities of stable conditions were detected in the absence or presence of the inoculant (Fig. 2B, Supplementary Table S4) is likely due to the inability of the community to grow given the nutrient-deprived environment. Interreplicate variability measured as the distance of each replicate to the centroid showed a moderate increase in variability with sampling time in the growing NatCom (Fig. 2C), probably caused by a still-evolving community, while this was only observed in the wheat rhizosphere of the stable condition, which could indicate that part of the stable NatCom population can grow using the root exudates as a carbon source [34, 36]. In addition, sampling time positively correlated with Bray-Curtis dissimilarities in growing NatCom samples, except in the inoculated wheat rhizosphere (Fig. 2D), while in the stable NatCom, a significant correlation with time was only observed in the rhizosphere of wheat.

These results indicate that nutrient availability must have been a major determinant for the different community trajectories between growing and stable NatComs. The successional changes that followed responded to the presence of wheat roots or the inoculation with *P. protegens* CHA0, except in the bulk conditions of the stable NatCom, where the slower rate of compositional changes across sampling times and no effect of the inoculant point to changes related to other phenomena such as competition for growth-limiting nutrients [82].

### Assembly processes are governed by nutrient availability but do not impact overall association networks

**Fig. 3:**
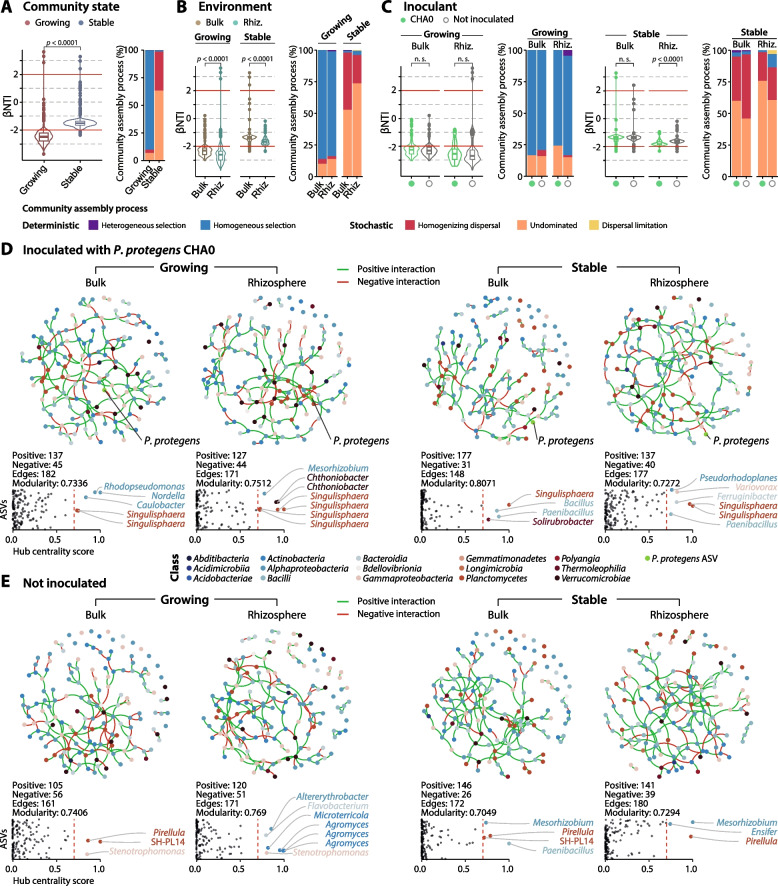
Community assembly processes of NatComs and ecological association upon establishment of *Pseudomonas protegens*. Community assembly processes dominating the bacterial communities in response to the different community states, growing or stable **A**; different environments, bulk soil or wheat rhizosphere **B**; or the inoculation with *P. protegens* CHA0 **C** were calculated based on deviation from null community models. Differences in beta-nearest taxon index (βNTI), together with Raup-Crick-based Bray-Curtis served to determine the community assembly process that dominated the samples (bar plots). Per tested condition, all sampling times were merged. The threshold for the |βNTI| = 2 is highlighted as horizontal red lines. Differences in βNTI are based on Wilcoxon rank-sum test. Not significant (n.s.): *p*-value > 0.05. Ecological association inference is based on sparse inverse covariance estimation among the top 150 ASVs (dots, coloured according to the bacterial class assignation) from growing or stable community states, in bulk soil or the wheat rhizosphere in response to the inoculation with *P. protegens* CHA0 **D** or not inoculated **E**. Samples from the sampling times 5, 7 and 9 days were combined. Positive and negative interactions are coloured in red or green, respectively. Hub centrality scores of each ASV (dots) in the networks are based on the scaled Kleinberg’s hub centrality score. ASVs with a hub score ≥ 0.7 were considered keystone taxa and coloured according to the bacterial class assignation

Processes driving the assembly of the communities were assessed based on entire-community null models. The community state alone (i.e. growing or stable) influenced the deviation from the null models (βNTI, Fig. 3A), with growing state being the most deviated. While the growing NatCom was dominated by deterministic processes, mainly homogeneous selection representing the 90%, the assembly of the stable NatCom was driven by stochastic processes (63% of undominated processes and 35% of homogenizing dispersal, Supplementary Table S5). Environment (bulk soil or wheat rhizosphere) was a significant factor in the model deviation (Fig. 3B), but inoculation was not, except in the case of the stable community rhizosphere (Fig. 3C). The attributed assembly processes were similar, but the fraction of undominated processes increased up to 74% in the stable community rhizosphere (Fig. 3B). Inoculation of *P. protegens* CHA0 reduced the proportion of homogeneous selection in favour of undominated processes in the stable community, both for the bulk soil and the wheat rhizosphere (Fig. 3C, Supplementary Table S5).

The dominance of homogeneous selection within the growing NatCom suggests that the addition of nutrients drives the community succession [63, 83], which was expected as the replicability of the NatCom is based upon this concept [46]. However, in the absence of a dominating force (i.e. nutrients), the stable NatCom drifted apart mainly due to stochastic processes of turnover and the absence of net growth [84]. Surprisingly, the proportion of undominated stochastic processes in the wheat rhizosphere of stable conditions was even higher than in the bulk soil and could be related to the observed interreplicate variability (Fig. 3B). This increased stochasticity is possibly linked to the early stages of rhizosphere microbiome formation, where root exudates serve as carbon sources for the bacteria [10], which may not be homogenously distributed in the vicinity of the roots. In addition, signalling molecules may enhance or impair the growth of certain taxa [16, 17], as would the plant immune responses [18]. Competition between the members of the community by multiple mechanisms and different levels of spatial exclusion [85] likely contribute as well to a complex mixture of processes that control the rhizosphere microbiome assembly.

The inference of taxa associations across treatments showed that communities overall were dominated by positive interactions, which were approximately three times more abundant than negative interactions (Fig. 3D, E). This finding contrasts with a current assumption that competition would be the dominant type of interaction between bacterial species [86]. However, positive correlations have been found to dominate in the rhizosphere microbiome [23]. Network modularity was almost independent of the community state, environment or the inoculation with *P. protegens* CHA0, ranging from 0.7049 in the bulk soil of not inoculated stable NatCom to 0.8071 in the bulk soil of the inoculated stable NatCom (Fig. 3D, E). This is in agreement with the modularity scores observed in other studies focusing on early rhizosphere microbiome and bulk soil assemblages [23]. Interesting differences, however, were found among the attributed keystone taxa in the different conditions and treatments. This attribution is based on the hub centrality score and indicated, for example *Singulisphaera* as a keystone taxon in all networks from samples inoculated with *P. protegens* CHA0, regardless of the community state or the environment. In non-inoculated samples, *Pirellula* had a dominating role except for the wheat rhizosphere of the growing community where other taxa such as *Agromyces* emerged as keystone. Both *Singulisphaera* and *Pirellula* are largely understudied members of the Planctomycetes class, usually ubiquitous in moderately acidophilic or mesophilic terrestrial habitats [87]. This difference may not be a direct effect of inoculation with *P. protegens* CHA0 but rather resulting from an indirect process affecting the interaction network of taxa. The reason is that the abundances of the two Planctomycetes members were not different in the presence or absence of the inoculant (see below) and might suggest that both genera exhibit similar niche exploitation in our microcosm conditions.

### The assembly of the NatCom-derived wheat rhizosphere microbiome selects specific taxa

Differential abundance analyses showed that the wheat rhizosphere environment produced a significant change in ASVs belonging to the Actinobacteriota, Bacteroidota, Firmicutes, Proteobacteria and Verrucomicrobiota phyla (Supplementary Fig. S5). The number of ASVs that were significantly enriched in the wheat rhizosphere was roughly two times higher in the stable NatCom compared to the growing condition, with 33 ASVs enriched in the wheat rhizosphere compared to 12 ASVs specifically enriched in the bulk soil inoculated with *P. protegens* CHA0 (and 29 compared to 15 ASVs in the non-inoculated systems, respectively). These changes reflect what would be expected for a community assemblage selecting a specialized rhizosphere microbiome [11, 88]. The enrichment of specific taxa in the wheat rhizosphere is also consistent with the previously observed increase in diversity, in both stable and growing conditions (Fig. 1D) and the divergence of rhizosphere communities from their bulk soil counterparts (Fig. 2A). Taxa specifically enriched in the wheat rhizosphere belong to known plant-associated genera, in particular *Flavobacterium*, *Paenibacillus*, the *Rhizobium* group, *Enterobacter* and *Pseudomonas* [89, 90], which are also found associated with the wheat rhizosphere [29, 88]. The specific enrichment of ASVs in the stable bulk soil (12 and 5 ASVs in the inoculated and non-inoculated conditions, respectively) might correspond to bacteria that are negatively affected by the plant (e.g. by allelopathic signalling molecules [16] or by plant immune responses [18]) or to bacteria that can secure limiting nutrients through different scavenging mechanisms, such as through the use of siderophores [82]. No specific bulk soil enrichment of ASVs was observed for the growing community condition.

*Pseudomonas* ASVs became differentially enriched depending on community states (Supplementary Fig. S5). *Pseudomonas* ASV1173 was enriched in the rhizosphere of the growing conditions, whereas *Pseudomonas* ASV1142 was enriched in the rhizosphere of the nutrient-limited conditions. The enrichment of both *Pseudomonas* occurred independently of the inoculation pattern. This suggests an additional component of selection of potential competitors to CHA0 depending on the state of the resident community.

### The establishment of *Pseudomonas protegens* alters the relative abundance of other NatCom Pseudomonas ASVs

**Fig. 4:**
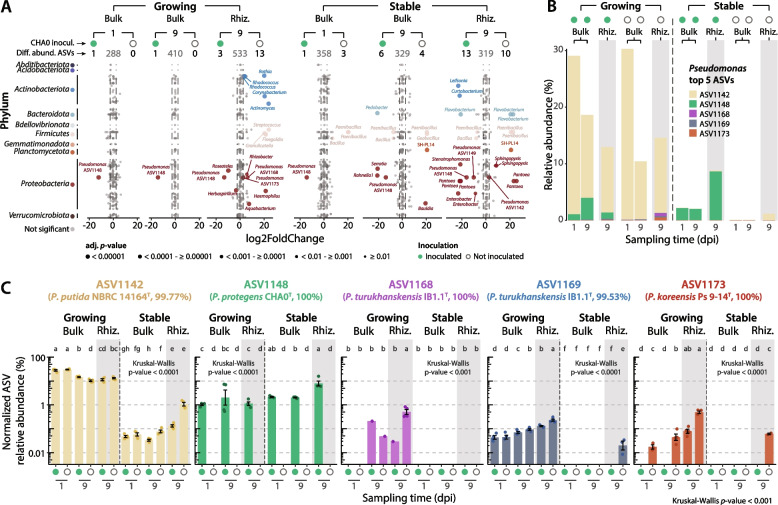
The establishment of *Pseudomonas protegens* causes discrete changes in growing or stable NatComs. **A** Differential abundance analyses comparing samples from the growing or stable community states, in the bulk soil or the wheat rhizosphere, inoculated with *P. protegens* CHA0 with those uninoculated at the initial or final sampling times (dpi, days post inoculation). The wheat rhizosphere (Rhiz.) was not sampled at the initial sampling time (1 dpi) due to limited biomass. Dots represent ASVs, with sizes according to adjusted (adj.) *p*-value and coloured according to their phylum assignation. The numbers of differentially abundant ASVs are indicated above (left, more abundant in the inoculated condition; centre and grey, not significantly different between conditions; right, more abundant in the uninoculated condition). ASVs with a |log_2_FoldChange| > 2.5 and a adj. *p*-value < 0.01 were considered significant. **B** Average relative abundance of the top five *Pseudomonas* ASVs at the initial (1 dpi) or final (9 dpi) sampling time. ASV1148 (green) corresponds to *P. protegens* CHA0. **C** CSS-normalized relative abundances of individual *Pseudomonas* ASVs across community states, environments, and inoculation patterns. Bars (±standard error) represent the average. Coloured dots represent individual replicates. Significance is based on Kruskal-Wallis rank-sum test with LSD post hoc analysis and *p*-value corrected by fdr. Different letters indicate significant differences between groups at *p*-value < 0.05. The type strain closest to the ASV is indicated below the ASV name (% sequence identity)

We further explored differential changes in the relative abundance of ASVs in the growing or stable NatComs in response to the inoculation with *P. protegens* CHA0. Overall, the number of changing ASV relative abundances was limited to a few taxa (Fig. 4A, Supplementary Table S6), consistent with previous studies in which the introduction of plant-beneficial inoculants resulted in small and transient changes in the overall rhizosphere microbiome (at least at this level of taxa resolution) [27, 91]. Most changes occurred in the wheat rhizosphere (Fig. 4A). Under nutrient-limited conditions, we observed a strain-specific selection by root exudates and/or the presence of the inoculant (e.g. *Paenibacillus*, *Flavobacterium* and *Pantoea*, Fig. S4). In the growing conditions, changes affected *Pseudomonas* ASVs different from the inoculant, i.e. ASV1168 and ASV1173, suggesting direct or indirect competition with the inoculant. The *Pseudomonas* genus is a highly diverse bacterial taxa, usually found in soils or associated with plants [92, 93], in which kin competition has been previously reported [22, 94].

A detailed exploration of the top five most abundant *Pseudomonas* ASVs (Fig. 4BC) revealed that in growing conditions, ASV1142 (assigned to *Pseudomonas putida*, Supplementary Table S7) dominates the *Pseudomonas* fraction of the communities, with up to *ca*. 30% of the relative abundance of the total microbiome at the first sampling time (Fig. 4B). However, under stable, nutrient-limited conditions, ASV1142 became scarce irrespective of *P. protegens* CHA0 inoculation, suggesting a nutrient-based growth limitation rather than competition with the inoculant (Fig. 4B). In contrast, the abundance of ASV1142 significantly increased in the wheat rhizosphere compared to stable bulk soil conditions (Fig. 4B, Supplementary Fig. S5). However, ASV1168, ASV1169 (both assigned to *P. turikhanskensis*) and ASV1173 (assigned to *Pseudomonas koreensis*, Supplementary Table S7) showed a reduced relative abundance when co-inoculated with CHA0 in the wheat rhizosphere of growing conditions (Fig. 4C), which may indicate competition. In fact, ASV1168, ASV1169, ASV1173 and CHA0 (ASV1148) all belong to different subgroups within the *Pseudomonas fluorescens* complex of species, largely known for their positive interaction with plants [92, 93], which make them likely dwellers of the wheat rhizosphere environment.

**Fig. 5:**
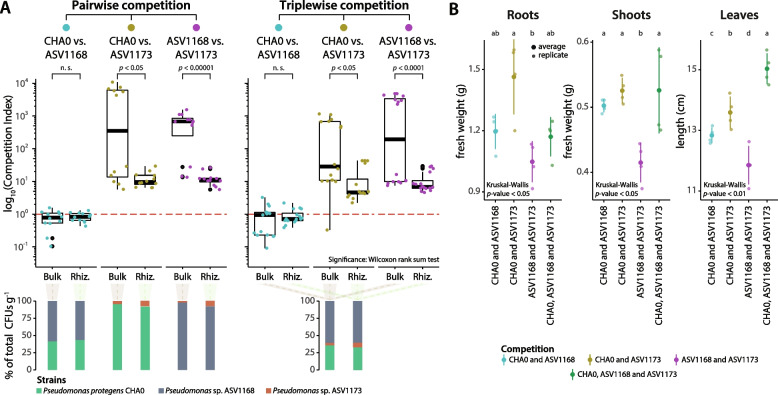
Competition of *Pseudomonas* strains in bulk soil or in the rhizosphere of wheat. **A** Pairwise (left, 1:1 ratio) or triplewise (right, 1:1:1 ratio) competition of *Pseudomonas protegens* CHA0, *Pseudomonas* sp. ASV1169 and *Pseudomonas* sp. ASV1173 in bulk soil or in the rhizosphere of wheat in growing conditions after 9 days without the NatCom. The competition index (CI) was calculated using the formula: CI = (CFUs of Sx at *t*9 days — CFUs of Sx at *t*0 days)/(CFUs of Sy at *t*9 days — CFUs of Sy at *t*0 days), where Sx and Xy denote the two strains being compared. Four technical replicates of four biological replicates (consisting of equal pools from four rhizospheres or bulk soil samples) were performed. The red dotted line indicates a competition where both strains would not be influenced by the presence of one another. Above this threshold, the competitor strain (CHA0 or ASV1168) outcompetes the other strain. Stacked bar plots below show the average percentage of CFUs recovered per tested strain. Significant pairwise differences are calculated according to Wilcoxon rank-sum test. Not significant (n.s.): *p*-value > 0.05. **B** Plant growth-promotion measurements in response to the inoculation of the two or three *Pseudomonas* strains compared in **A**. Root﻿ and shoot fresh weights were calculated within the four pools of four rhizospheres or shoots per replicate. The length of leaves was measured individually and averaged by the pools of four plants shown for the other parameters. Significant differences were calculated using the Kruskal-Wallis rank-sum test with LSD post hoc analysis and *p*-value corrected by fdr. Different letters indicate significant differences between groups at *p*-value < 0.05

To verify this further, we conducted competition experiments (in absence of resident NatComs) between the three closely related *Pseudomonas* strains (Fig. 4, Supplementary Fig. S6), i.e. *P. protegens* CHA0 (ASV1148), and *Pseudomonas* sp. ASV1168 and ASV1173, both in pairs and in triplets. The results showed that CHA0 and ASV1168 are able to coexist and reach similar proportions either in bulk soil or in the wheat rhizosphere (Fig. 5A, Supplementary Table S8). In contrast, both strains were able to outcompete *Pseudomonas* sp. ASV1173 in pairs, to a higher extent in the bulk soil than in the wheat rhizosphere (Fig. 5A). Co- or triple inoculation with *P. protegens* CHA0 increased the shoot and root biomass of the wheat plants (Fig. 5B), but the response was higher with the CHA0-ASV1173 inoculation than with the CHA0-ASV1168 pair (Fig. 5B). This could be due to the displacement of ASV1173, thus increasing the abundance of *P. protegens* CHA0 (Fig. 5A) capable of exerting this growth-promoting effect.

The results obtained from these competition assays contrast with those obtained in the presence of a resident soil microbiome, where an order of magnitude higher normalized relative abundance of *P. protegens* CHA0 compared to ASV1168 was achieved in the wheat rhizosphere or bulk soil within the growing NatCom (Fig. 4C). However, in direct competition, in the absence of the NatCom, the two strains established at similar numbers. Furthermore, ASV1168 showed a significantly reduced abundance when exposed to *P. protegens* CHA0 in the wheat rhizosphere (Fig. 4C). Several factors could explain these differences, including the tagging of the strains (although no difference in growth rates compared to their wild types was observed, Supplementary Fig. S6), indirect fine-tuning interactions with other members of the microbiome [23, 29], or they might respond to different spatial colonization patterns on the plant roots [95], highlighting the importance of structurally complex environments, such as the wheat rhizosphere or the soil matrix for the prevalence of certain bacteria.

## Conclusions

The proliferation of plant-beneficial bacterial inoculants in rhizosphere microbiomes remains poorly understood due to the highly complex nature of the rhizosphere and soil environments, and fully reductionist approaches that focus on bacteria-bacteria interactions or synthetic communities only provide limited insight. Nonetheless, the investigation of the interplay of an inoculant with a species-rich microbiome and a key environmental factor such as nutrient availability as undertaken in the present study might broaden our understanding of inoculant establishment strategies.

We studied the effect of the plant-beneficial strain *Pseudomonas protegens* CHA0 when introduced into a soil natural, species-rich bacterial community (NatCom) [46] established in bulk soil and upon its assembly in the rhizosphere of wheat. We explored two community states, based on nutrient availability, a growing NatCom produced by community dilution and addition of new nutrients and a stable, nutrient limited NatCom in a stationary growth state. Our results are in line with the concept that access to nutrients is a major determinant of microbiome composition and structure, diversity, and assembly processes [96, 97]. The *P. protegens* inoculant was able to establish at a relatively small abundance within the microbiomes in all conditions tested, but this peaked in the wheat rhizosphere under stable conditions, which also reduced the diversity of the rhizosphere microbiome. This supports the concept that in a nutrient-limited environment, the plant rhizosphere can provide a niche available to specific taxa, due to root-exuded nutrients and signalling compounds [34, 36], thus supporting proliferation of adapted inoculants. The changes in the microbiomes over time showed that under growing conditions, the environment and the inoculation pattern lead to diverging trajectories. The lack of convergence along sampling times evidenced an early microbiome assembly process [23, 81]. Whether microbiome convergence will be attained or if the inoculant will also persist for a longer period remains to be studied. In addition, the changes observed in the growing condition were mainly explained by homogeneous selection as the main deterministic assembly process, independent of environment (bulk soil or wheat rhizosphere) or inoculation pattern, emphasizing nutrient availability as the dominant force driving communities to the observed compositions. Conversely, under stable, nutrient-limited conditions, we found microbiome divergence only in the wheat rhizosphere while observing no differences in the inoculation regime and a reduced speed of microbiome drift in the stable bulk soil. Furthermore, the lack of a common driving force in this condition fits a more complex scenario in which various processes may explain the observed microbiome composition. Notably, the rhizosphere effect might be amplified, being the only source of available nutrients in the stable community, where other processes, such as specialized scavenging mechanisms, might be more prevalent [82]. Indeed, the rhizosphere environment produced changes in certain ASVs, mainly from the Actinobacteria, Bacteroidota, Firmicutes and Proteobacteria phyla, which were mostly enriched in stable conditions. Association inference of taxa showed that communities were dominated by positive interactions while modularity remained constant across samples. Nonetheless, the presence of different keystone taxa depending on the inoculation pattern suggests altered community networks. In fact, the introduction of *P. protegens* CHA0 changed a discrete number of ASVs mostly affecting the rhizosphere of both growing and stable conditions. This implies that the introduction of the inoculant does not radically alter the composition of the rhizosphere or bulk soil microbiome, as also observed by other authors using different *Pseudomonas* inoculants [26, 27]. The observed changes involve nevertheless a reduced prevalence of specific *Pseudomonas* ASVs, commonly associated with plant hosts [92, 93], making them likely competitors in the rhizosphere environment. Direct competition of the inoculant with two *Pseudomonas* strains isolated from the NatCom revealed that competition in the bulk soil or the rhizosphere environment only partially explains the observations at the microbiome level. This suggests that other factors may be at play, including a more complex network of interactions across the microbiome members or a different spatial distribution across roots or the soil interface [19, 23], which may play an important role in defining the plant-rhizosphere microbiome.

The approach followed in the present study, using a species-rich natural soil bacterial community in a structurally complex environment, allowed us to demonstrate that the niche created by the wheat rhizosphere allows the proliferation of *P. protegens* CHA0, which also competes efficiently with closely related bacteria.

### Supplementary Information

**Additional file 1:** **Supplementary Fig. S1.** Differences in the relative abundance of the main bacterial Classes across samples identified in this study. **Supplementary Fig. S2.** Alpha diversity indexes across samples and timepoints. **Supplementary Fig. S3.** Plant growth measurements across sampling times. **Supplementary Fig. S4.** Bray-Curtis dissimilarities across samples. **Supplementary Fig. S5.** Differential abundance analyses comparing samples from the bulk soil to the wheat rhizosphere, inoculated or not with *Pseudomonas protegens* CHA0. **Supplementary Fig. S6.** Growth differences between wild type (wt) and tagged *Pseudomonas* strains.

**Additional file 2:** **Supplementary Table S1.** Number of sequences per sample across the different processing steps. **Supplementary Table S2.** Relative abundance of bacterial classes. **Supplementary Table S3.** Alpha diversity indexes across samples. **Supplementary Table S4.** PERMANOVA test of Bray-Curtis dissimilarities across samples. **Supplementary Table S5.** Community assembly processes per community state, environment or inoculation. **Supplementary Table S6.** Differential abundance of ASVs between all conditions tested. **Supplementary Table S7.** Top five most abundant *Pseudomonas* ASVs, closest relatives and 16S rRNA sequence similarity to isolated strains. **Supplementary Table S8.** CFUs and competition index between the three *Pseudomonas* used in this work.

## Data Availability

All data generated and analysed in this study is publicly available in the NCBI Sequence Read Archive (RSA) under the BioProject accession number PRJNA948847 or included as supplementary information. The original R script used in this study, the metadata file, raw ASV sequences and the taxonomy table are publicly available in GitHub (https://github.com/dgarrs/https://github.com/dgarrs/Pprotegens_proliferation_NatComs).

## Abbreviations

Adj.: *Adjusted*
ASVs: Amplicon sequence variants
CFUs: Colony-forming units
CHA0: *Pseudomonas protegens* CHA0
CI: Competition index
CSS: Cumulative sum scaling
dpi: Days post inoculation
FC: Fold change
fdr: False discovery rate
GAM: General additive model
Gm: Gentamycin
LSD: Fisher’s least significant difference
ML: Maximum-likelihood
MM: Mineral medium
NA: Nutrient agar
NatCom: Soil natural bacterial community
NMDS: Nonmetric multidimensional scaling
NYB: Nutrient yeast broth
OD: Optical density
PD: Phylogenetic diversity
PERMANOVA: Permutational multivariate analyses of variance
RC_bray_: Raup-Crick-based Bray-Curtis
Rhiz.: Rhizosphere
Rif: Rifampicin
SE: Soil extract
SPIEC-EASI: Sparse inverse covariance estimation for ecological association inference
βMNTD: Beta-mean nearest taxon distance
βNTI: Beta-nearest taxon index
