# Supplementary material for: Changes in structure and assembly of a species-rich soil natural community with contrasting nutrient availability upon establishment of a plant-beneficial Pseudomonas in the wheat rhizosphere
Source: Microbiome. 2023 Sep 29;11:214. doi: 10.1186/s40168-023-01660-5 (PMC10540321; doi:10.1186/s40168-023-01660-5)
Supplement: Supplementary file 2 — Additional file 1: Supplementary Fig. S1. Differences in the relative abundance of the main bacterial Classes across samples identified in this study. Supplementary Fig. S2. Alpha diversity indexes across samples and timepoints. Supplementary Fig. S3. Plant growth measurements across sampling times. Supplementary Fig. S4. Bray-Curtis dissimilarities across samples. Supplementary Fig. S5. Differential abundance analyses comparing samples from the bulk soil to the wheat rhizosphere, inoculated or not with Pseudomonas protegens CHA0. Supplementary Fig. S6. Growth differences between wild type (wt) and tagged Pseudomonas strains. [file 40168_2023_1660_MOESM1_ESM.pdf]

Changes in structure and assembly of a species-rich soil natural community with contrasting nutrient availability upon establishment of a plant-beneficial *Pseudomonas* in the wheat rhizosphere

– Supplementary information –

Daniel Garrido-Sanz[1#], Senka Čaušević[1], Jordan Vacheron[1], Clara M. Heiman[1], Vladimir Sentchilo[1], Jan Roelof van der Meer[1], Christoph Keel[1#]

[1] Department of Fundamental Microbiology, University of Lausanne, CH-1015 Lausanne, Switzerland

[#] **Corresponding authors:** Daniel Garrido-Sanz (daniel.garridosanz@unil.ch) and Christoph Keel (christoph.keel@unil.ch)

Contents

Supplementary Fig. S1

Supplementary Fig. S2

Supplementary Fig. S3

Supplementary Fig. S4

Supplementary Fig. S5

Supplementary Fig. S6

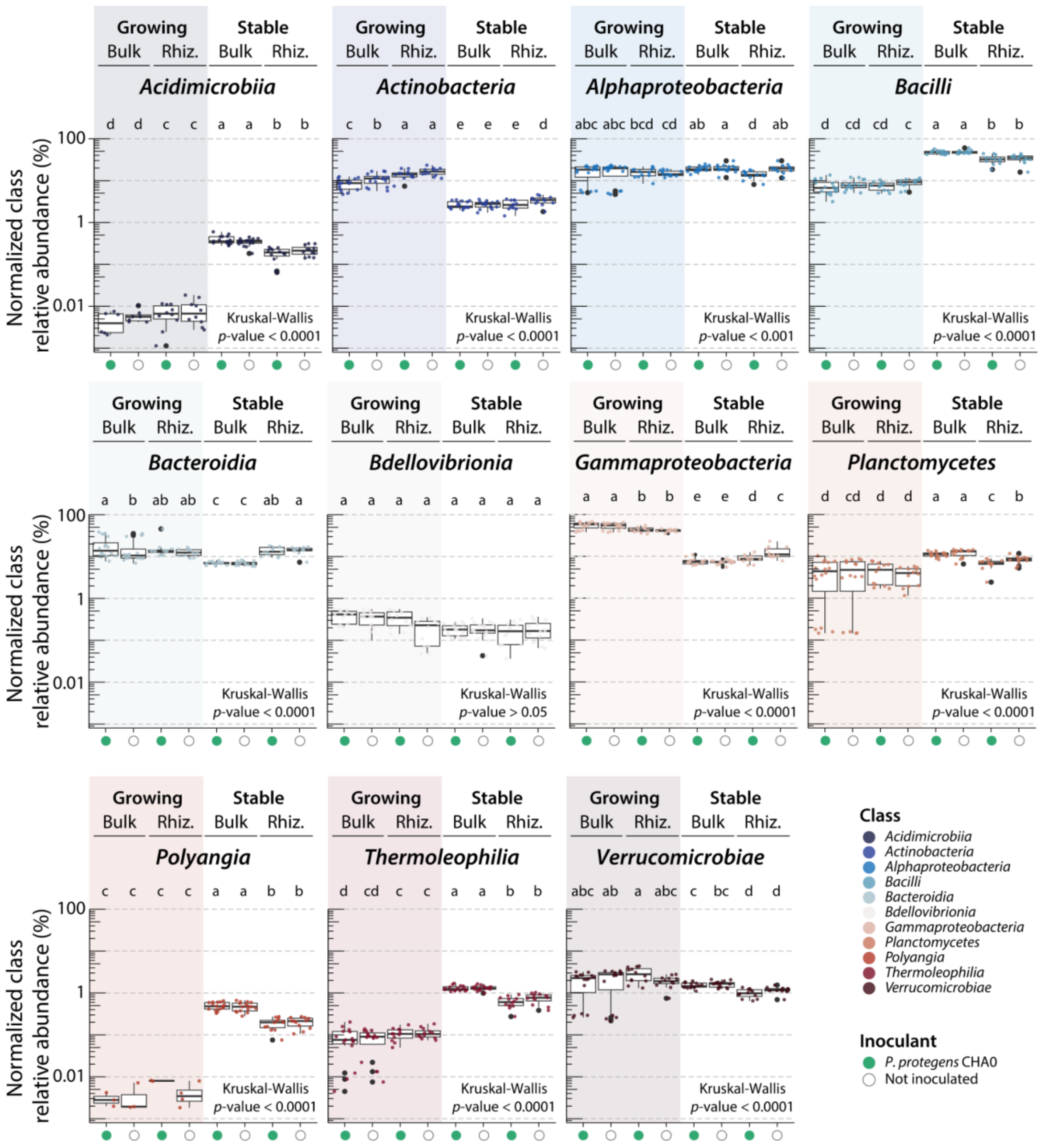

Supplementary Fig. S1. Differences in the CSS-normalized relative abundance of the main bacterial classes across samples identified in this study. Differences are indicated by community state (growing or stable), environment (bulk soil or wheat rhizosphere), or the inoculation with *Pseudomonas protegens* CHA0 (green dots) or not inoculated (empty dots). For *Gammaproteobacteria*, *P. protegens* ASV was removed to avoid an artificial inflation of the class relative abundance. Significance based on Kruskal-Wallis rank sum test with LSD post hoc analysis and *p*-value corrected by fdr. Different letters indicate significant differences between groups at *p*-value < 0.05.

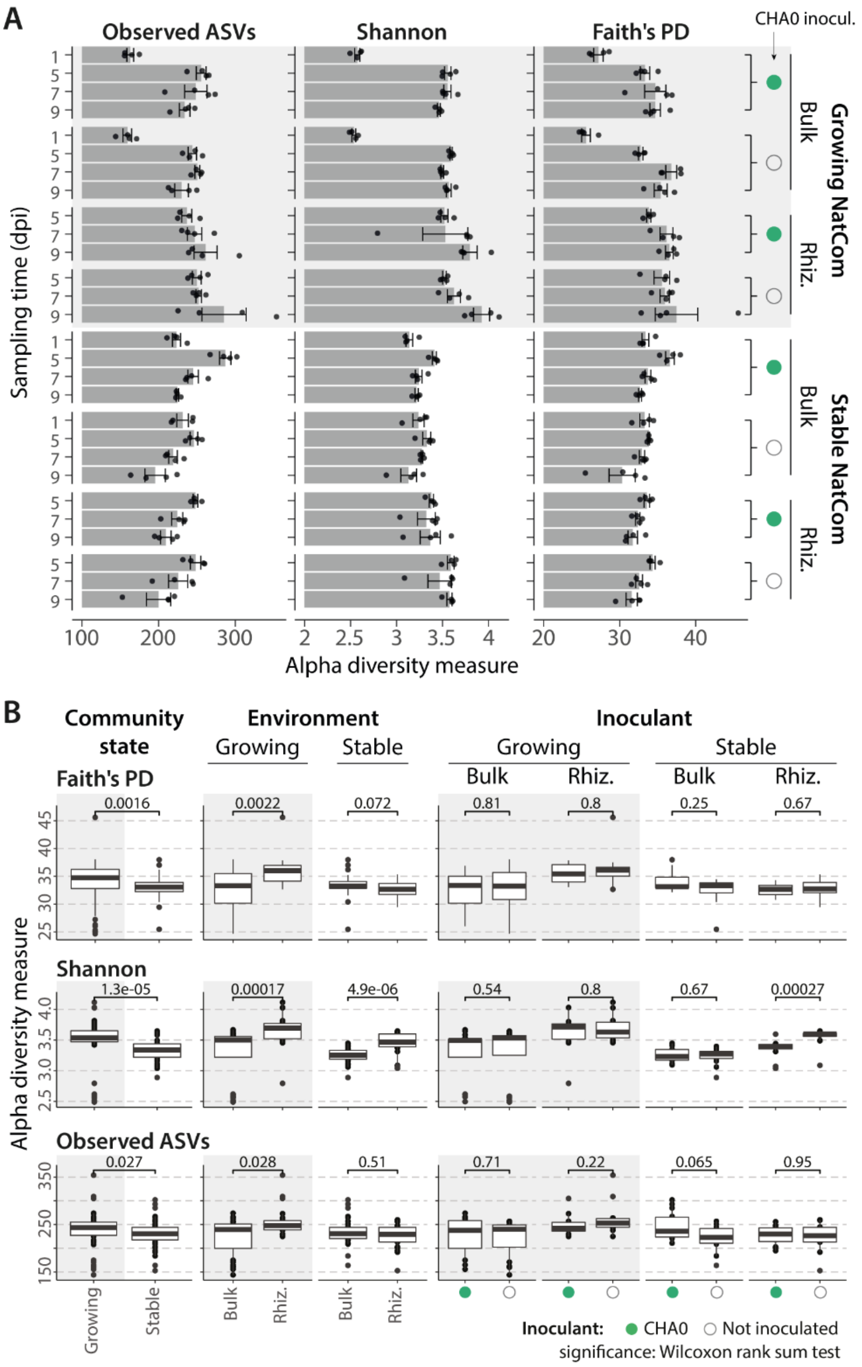

Supplementary Fig. S2. Alpha diversity indexes across samples and timepoints. (**A**) The number of observed ASVs, the Shannon diversity index and the Faith's phylogenetic diversity (PD) were calculated per replicate and represented as bar plots (mean values ± standard error). Dots indicate individual replicate values. (**B**) Differences in diversity are indicated by community state (growing or stable), environment (bulk soil or wheat rhizosphere), or the inoculation with *Pseudomonas protegens* CHA0 (green dots) or not inoculated (empty dots). The significance according to Wilcoxon rank sum test and *p*-values is indicated per comparison. Samples from the growing community state are highlighted with a grey background.

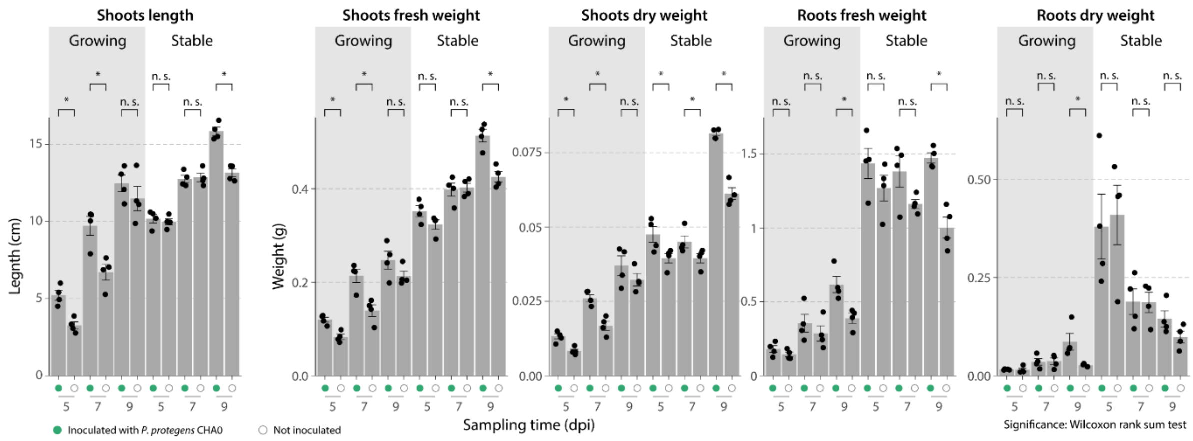

Supplementary Fig. S3. Plant growth measurements across sampling times. Individual samples (dots) are calculated by four pooled plant shoots or roots, respectively. Mean values (± standard errors) are represented as bars. Significance between samples inoculated with *P. protegens* CHA0 (green dots) or not inoculated (empty dots) is calculated with the Wilcoxon test. Not significant (n. s.): *p*-value > 0.05; *: *p*-value < 0.05. Samples from the growing community state are highlighted with a grey background.

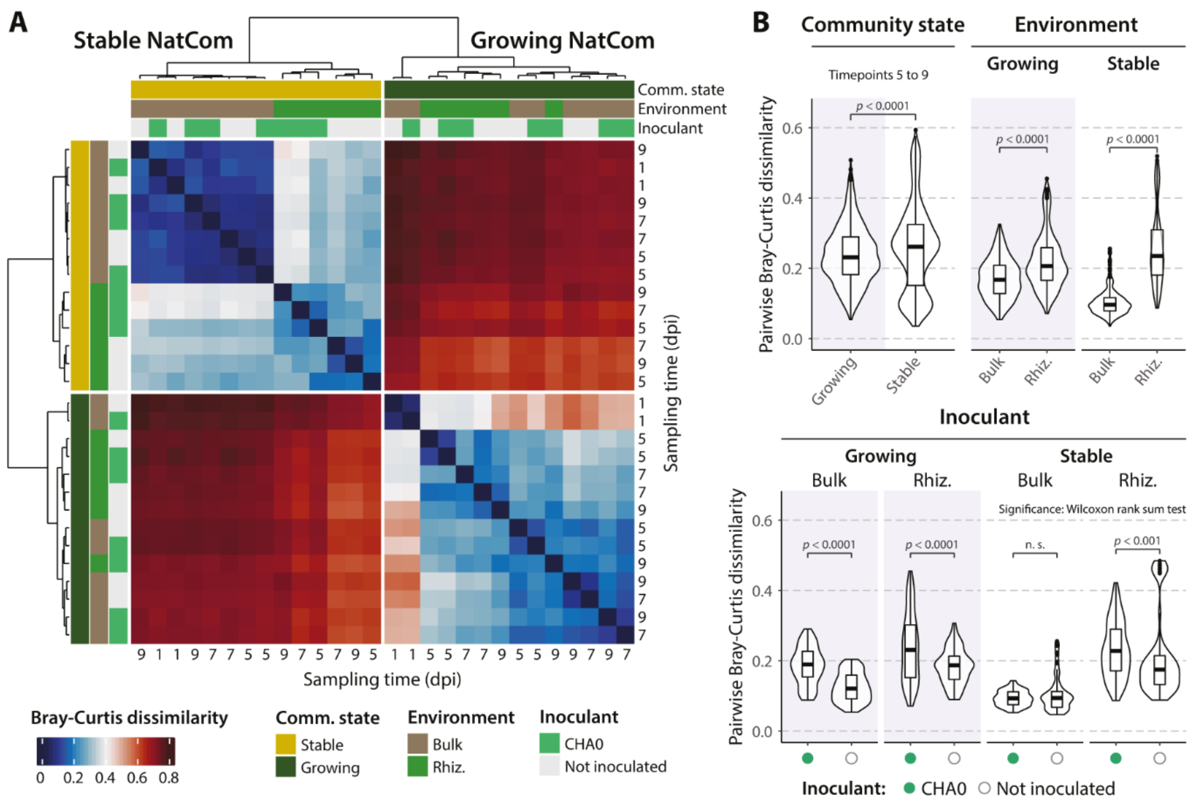

Supplementary Fig. S4. Bray-Curtis dissimilarities across samples. (**A**) Clustering analysis of Bray-Curtis dissimilarities across samples and sampling times for stable and growing NatComs. The four replicates per sampling time and condition, i.e., community state (growing or stable), environment (bulk soil or wheat rhizosphere), or inoculated with *P. protegens* CHA0 or not inoculated, were combined (sum) prior to dissimilarity calculation. dpi, days post inoculation. (**B**) Differences in the pairwise Bray-Curtis dissimilarity in community states (growing or stable), environments (bulk soil or wheat rhizosphere (Rhiz.)) or the inoculation with *P. protegens* CHA0 (green dots) or not inoculated (empty dots). The first sampling time (1 dpi) was not considered. Significance based on the Wilcoxon rank sum test. Not significant (n. s.): p-value > 0.05.

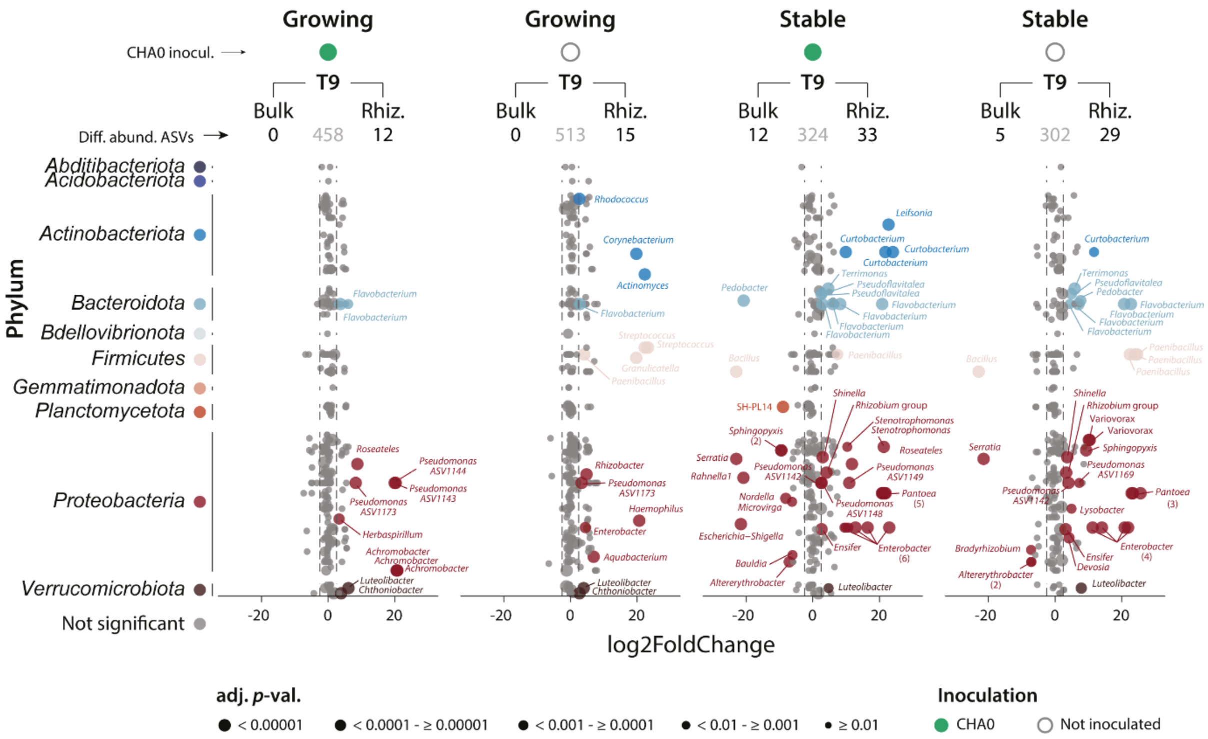

Supplementary Fig. S5. Differential abundance analyses comparing samples from the bulk soil to the wheat rhizosphere, inoculated or not with *Pseudomonas protegens* CHA0. Dots represent ASVs, size according to adjusted *p*-value and coloured according to their Phylum. The number of differentially abundant ASVs are indicated above (left, more abundant in the bulk soil; centre and grey, not significantly different between conditions; right, more abundant in the wheat rhizosphere). ASVs with a |log2FoldChange| > 2.5 and a adj. *p*-value < 0.01 were considered differentially abundant.

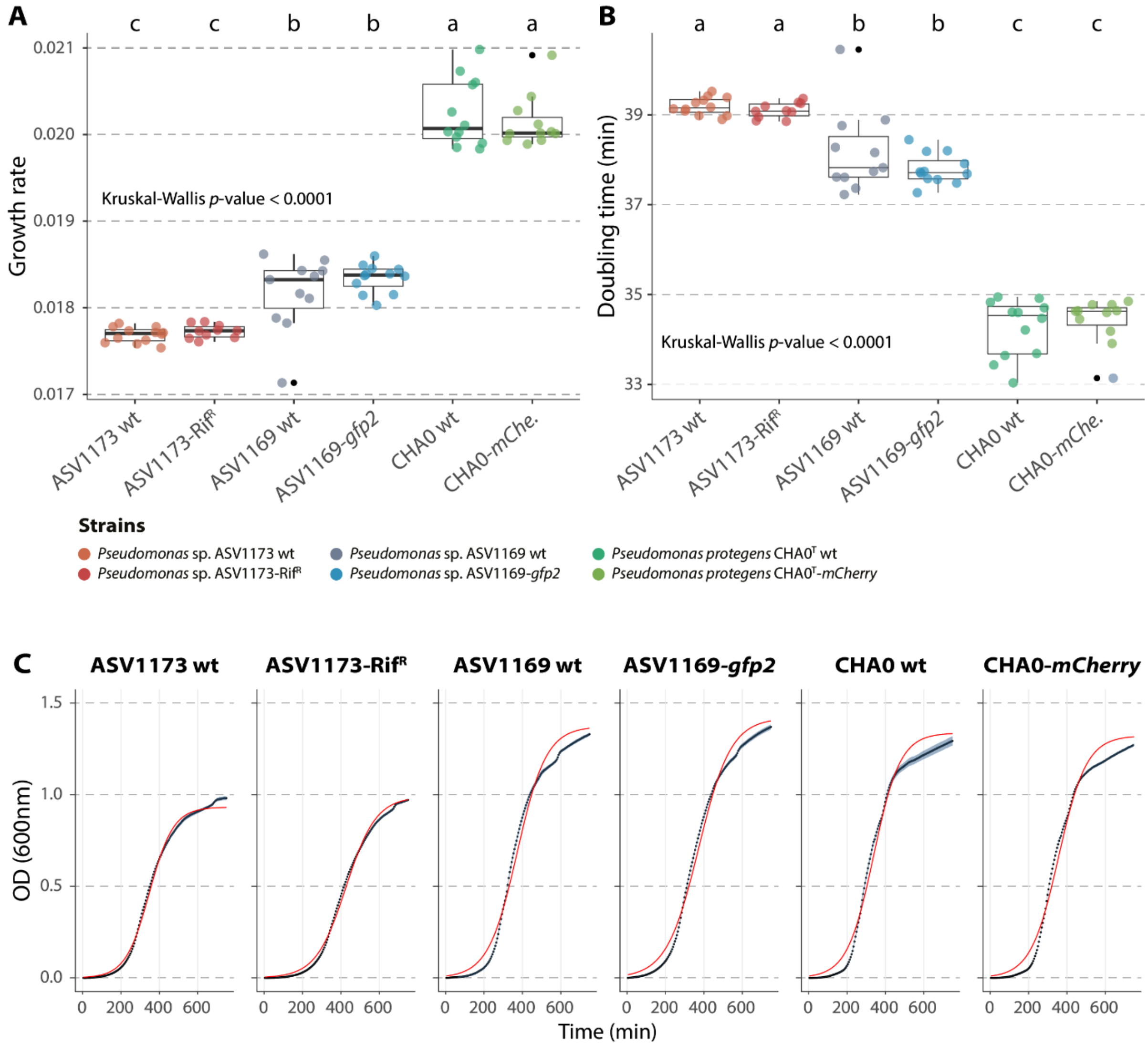

Supplementary Fig. S6. Growth differences between wild type (wt) and tagged *Pseudomonas* strains. (**A**) Growth rate and (**B**) doubling time obtained from the growth curves of 12 technical replicates. Different letters indicate significant differences between groups at a *p*-value < 0.05. Significance based on Kruskal-Wallis rank sum test with LSD post hoc analysis and *p*-value corrected by fdr. (**C**) Individual curves of the different strains used. Black dots represent mean values. Blue shadows represent the standard error from the 12 technical replicates. The logistic fitting curve is indicated in red. Strains were grown in NYB medium.
